# Lamin A/C missense variants: from discovery to functional validation

**DOI:** 10.1038/s41525-021-00266-w

**Published:** 2021-12-03

**Authors:** Julieta Lazarte, Robert A. Hegele

**Affiliations:** 1 Department of Medicine, Schulich School of Medicine and Dentistry, Western University, London, ON Canada; 2 Department of Biochemistry, Schulich School of Medicine and Dentistry, Western University, London, ON Canada; 3 Robarts Research Institute, Schulich School of Medicine and Dentistry, Western University, London, ON Canada

**Keywords:** Molecular medicine, Disease genetics

## Abstract

Rare variants in the *LMNA* gene encoding nuclear lamin A/C are causal for more than a dozen diverse mendelian disorders. Defining the functional consequences of *LMNA* variants has been challenging given the pleiotropy of gene functions and potential pathogenic mechanisms. It is essential to develop trustworthy, scalable and rapidly deployable in vitro assays of function to enable timely assessment of missense variants that are being uncovered by high throughout next-generation sequencing.

Nuclear lamins play multifunctional roles in cell biology. They provide structural support for the nuclear contents, help to govern nuclear sensing and mechanotransduction, and simultaneously regulate gene expression by organizing and interacting with chromatin and transcription factors^1–3^. Nuclear lamins A and C are encoded by the *LMNA* gene on chromosome 1q22 and are structurally composed of coils 1A, 1B and 2 which make up the α-helical rod domain, an immunoglobulin (Ig)-fold which makes up the Ig tail, and a nuclear localization signal (NLS) which separates the coiled-coil and Ig domains^4^. Given the complex nature of the *LMNA* gene and the diverse roles played by the encoded lamin isoforms, rare pathogenic variations have been found to underlie more than a dozen heterogenous largely autosomal dominant conditions and syndromes that are collectively referred to as “laminopathies”^4,5^. It is thus not surprising that different *LMNA* mutations will impact different functions and roles of lamins, such as nuclear morphology, integrity, mechanosensing and mechanotransduction, direct interaction with transcription factors, altered functionality of mutant pre-lamins, altered processing of lamin and altered interaction with chromatin, which collectively help begin to explain the wide range of associated clinical phenotypes^4–10^.

Laminopathies due to rare *LMNA* variants affecting lamin A/C can be sorted into disorders that affect specific tissues, including cardiac muscle (dilated cardiomyopathy), skeletal muscle (Emery–Dreifuss muscular dystrophy), adipose tissue (familial partial lipodystrophy type 2), peripheral nerves (Charcot-Marie-Tooth disorder type 2B1), as well as systemic disorders (progeria syndromes)^1,4,5,10,11^. Over the last two decades, >600 *LMNA* missense variants associated with various diseases have been deposited in the ClinVar database. The sheer diversity of affected tissues, organ systems, and distinctive syndromes is exceptional for a single gene. This inherent biological complexity, in turn, poses unique challenges when attempting to ascribe pathogenicity or dysfunction to any newly discovered variant including missense variants of uncertain significance (VUSs), which are being revealed at an accelerating rate and comprise a substantial proportion of archived variants for *LMNA*, as they are for most other genes.

For two decades investigators have endeavoured to link attributes of specific *LMNA* variants to precise functional consequences that in turn might mechanistically explain the varied clinical presentations. For instance, an attribute as simple as the position of a pathogenic *LMNA* missense variant relative to the coding sequence for the NLS predicts tissue involvement: variants upstream of NLS are 8-times more likely to be associated with cardiomyopathy and skeletal myopathy than downstream variants, which are instead more likely to be associated with lipodystrophy and progeria^12^. The majority of the pathogenic mutations in *LMNA* identified so far have been linked to an aggressive type of inherited cardiomyopathy that includes conduction disorders, arrhythmias, ventricular dysfunction, and heart failure^1^. However, more work is required to increase the rigour of functional characterization and understanding of the cellular biology that links a particular rare *LMNA* variant to a specific clinical phenotype.

To address the challenges of testing functionality of *LMNA* variants including VUSs, Anderson et al. in this issue of *npj Genomic Medicine* evaluated 178 missense variants located in different structural and functional domains of lamin A/C via a high-throughput approach^13^. They primarily used two mammalian overexpression models, employing human embryonic kidney (HEK) 293 cells and mouse C2C12 myoblasts. They evaluated nuclear aggregation as their key functional read-out, both because misfolding and aggregation are important mechanisms in other dominant disorders, but also because past studies of individual lamin A/C variants have provided evidence of aberrant aggregation^8^. Also, by demonstrating that their assay is reliable, robust and scalable, they set the stage for future applications, including potential incorporation of such an assay into the evidence-gathering pipeline for the dysfunctionality criterion within the guidelines classification of pathogenicity.

The specific read-outs of interest were lamin A aggregation in HEK 293 and C2C12 cells, and manually counted from >100 cells in triplicate, stratified by variants within either the coiled domains or Ig like domain. The authors also evaluated lamin A aggregation in induced pluripotent stem cell (iPSC)-cardiomyocytes isolated by flow cytometry to validate a cell-specific mechanism for variants associated with cardiomyopathy. The suite of experiments included co-expression of selected variants with wild-type lamin A/C to evaluate a potential dominant negative effect for variants showing dominant inheritance. They complemented this work with in silico predictions of aberrant folding and finally observed correlation between in vitro findings and clinical phenotypes.

From these experiments, Anderson *et al*. showed that lamin A aggregation abnormalities are the main read-out for missense variants that are associated with skeletal and cardiac myopathies with generally good correspondence between findings across cell types. They found that 84%, 52%, 35 and 17% of variants associated with skeletal myopathies, cardiomyopathies, lipodystrophy and progeria showed abnormal aggregation, suggesting that aggregation abnormalities represent a functional phenotype that is more specific for myopathic laminopathies compared with other types of laminopathies. They also showed that aggregation-prone variants in the Ig domain are generally associated with greater protein instability and misfolding. Finally, they observed non-random concordance between these in vitro findings and clinical manifestations.

The approach and findings seem to represent a step forward in making sense of the complexity of human genetic variation in *LMNA* and its relationship to disease. In vitro functional analysis is weighed heavily when gauging pathogenicity of variants, both in classical human molecular genetic research and now with guidelines like those from the American College of Medical Genetics and Genomics^14^. Placing priority on functional characterization may be justified if the surrogate assay actually reflects a relevant molecular or cellular process that is important for pathogenesis^15^. But making such a link is complicated in the case of *LMNA*, given the vast underlying functional pleiotropy. Nonetheless, pleiotropy itself should not be a deterrent, as it is the rule rather than the exception underlying the associations between many human genes and disease phenotypes^16^.

The assays reported by Anderson et al. seem to be reproducible and scalable, and have the potential to provide useful adjunctive evidence for attributing pathogenicity to newly discovered specific missense variants. Ultimately, all in vitro functional analysis have limitations, however there would be practical advantages to timely access to a reliable result that makes sense in the context of all the other evidence, such as in silico modelling, allele frequency, co-segregation with phenotype, evolutionary conservation, etc. However, functional analysis is just one piece of evidence^17^. Furthermore, if the assay is somehow incorrect, misleading, unreliable or not really representative of the true pathogenesis, applying it prematurely in a clinical diagnostic pipeline might introduce errors into pathogenicity attribution, which benefits no one^18^. But these hazards have always existed in human mutation discovery when the work shifts to designing and interpreting in vitro functional assays. In the current milieu in which clinical next-generation sequencing is uncovering human missense variants at a torrential rate—many of which are VUSs—the concomitant development of reliable high throughput functional assays that can reliably upgrade (or downgrade) the pathogenicity status of a VUS is incredibly important. In the case of cardiomyopathic and skeletal myopathic variants in *LMNA*, the approach reported by Anderson and colleagues sketches out one possible starting point.
